# Combining proteomics and transcriptome sequencing to identify active plant-cell-wall-degrading enzymes in a leaf beetle

**DOI:** 10.1186/1471-2164-13-587

**Published:** 2012-11-01

**Authors:** Roy Kirsch, Natalie Wielsch, Heiko Vogel, Aleš Svatoš, David G Heckel, Yannick Pauchet

**Affiliations:** 1 Entomology department, Max Planck Institute for Chemical Ecology, Hans-Knöll-Str. 8, 07745, Jena, Germany; 2 Mass spectrometry Research Group, Max Planck Institute for Chemical Ecology, Hans-Knöll-Str. 8, 07745, Jena, Germany

## Abstract

**Background:**

The primary plant cell wall is a complex mixture of polysaccharides and proteins encasing living plant cells. Among these polysaccharides, cellulose is the most abundant and useful biopolymer present on earth. These polysaccharides also represent a rich source of energy for organisms which have evolved the ability to degrade them. A growing body of evidence suggests that phytophagous beetles, mainly species from the superfamilies Chrysomeloidea and Curculionoidea, possess endogenous genes encoding complex and diverse families of so-called plant cell wall degrading enzymes (PCWDEs). The presence of these genes in phytophagous beetles may have been a key element in their success as herbivores. Here, we combined a proteomics approach and transcriptome sequencing to identify PCWDEs present in larval gut contents of the mustard leaf beetle, *Phaedon cochleariae*.

**Results:**

Using a two-dimensional proteomics approach, we recovered 11 protein bands, isolated using activity assays targeting cellulose-, pectin- and xylan-degrading enzymes. After mass spectrometry analyses, a total of 13 proteins putatively responsible for degrading plant cell wall polysaccharides were identified; these proteins belong to three glycoside hydrolase (GH) families: GH11 (xylanases), GH28 (polygalacturonases or pectinases), and GH45 (β-1,4-glucanases or cellulases). Additionally, highly stable and proteolysis-resistant host plant-derived proteins from various pathogenesis-related protein (PRs) families as well as polygalacturonase-inhibiting proteins (PGIPs) were also identified from the gut contents proteome. In parallel, transcriptome sequencing revealed the presence of at least 19 putative PCWDE transcripts encoded by the *P. cochleariae* genome. All of these were specifically expressed in the insect gut rather than the rest of the body, and in adults as well as larvae. The discrepancy observed in the number of putative PCWDEs between transcriptome and proteome analyses could be partially explained by differences in transcriptional level.

**Conclusions:**

Combining proteome and transcriptome sequencing analyses proved to be a powerful tool for the discovery of active PCWDEs in a non-model species. Our data represent the starting point of an in-depth functional and evolutionary characterization of PCWDE gene families in phytophagous beetles and their contribution to the adaptation of these highly successful herbivores to their host plants.

## Background

Plant cells are encased within the primary cell wall, a complex of polysaccharides and proteins. The primary cell wall is typically composed of two main polysaccharide networks: one is made of cellulose microfibrils linked together with a hemicellulose matrix, and the other, made of pectins, is a complex heteropolysaccharide that hydrates and further cements the primary cell wall matrix [1,2]. Pectins represent 35% of primary cell wall polysaccharides in dicots and non-graminaceous monocots [3], hemicellulose accounts for 20-30% and the rest corresponds to cellulose [2]. The primary cell wall plays a role in various fundamental physiological processes such as plant growth and development, morphogenesis, cell signaling, cell-cell interactions, and protection against pathogens, dehydration and other environmental factors [1,3].

Some microorganisms, especially phytopathogenic bacteria and fungi [4,5], as well as plant parasitic nematodes [6,7] are very efficient in degrading plant cell wall polysaccharides either to use them as nutrients for their own growth or to get access to plant cells. These organisms secrete an impressive arsenal of enzymes specifically targeting plant cell wall polysaccharides, referred to here as plant cell wall degrading enzymes or PCWDEs. Among these, polygalacturonases, pectin methylesterases and pectin lyases degrade the pectin network, whereas various endo- and exoglucanases target the cellulose/hemicellulose network [4,5,7].

In insects, endogenous cellulase genes are apparently absent from the genome of model insects such as *Drosophila melanogaster* or *Bombyx mori*. Cellulase genes are also absent from the recently sequenced genomes of two butterflies, the Monarch *Danaus plexippus* and the Postman *Heliconius melpomene*, whose caterpillars are herbivorous feeding on milkweed and passion flower respectively [8,9]. On the other hand, the association between wood-feeding termites and their symbiotic protists offers a textbook example of cellulose digestion in insects, in which not only the protists provide cellulose-degrading enzymes but also the insect itself [10,11]. Furthermore, endogenous cellulase genes have also been discovered in other xylophagous insects, for example, cerambycid beetles [12-16]. Recently, a single endogenous cellulase from the glycoside hydrolase family 9 (GH9) has been identified and functionally characterized from the genome of the model beetle *Tribolium castaneum*[17]. PCWDEs other than cellulases, such as polygalacturonases, pectin methylesterases, mannanases and xylanases, have also been identified in insects, some of them recently, mainly in plant bugs [18,19] and phytophagous beetles [20-26]. Finally, our recent transcriptome survey revealed that putative PCWDEs are encoded by diverse and complex multigene families in phytophagous beetles from the superfamilies Chrysomeloidea and Curculionoidea, mainly in species of leaf beetles (Chrysomelidae), weevils and bark beetles (both Curculionidae) [27]. The presence of such genes in herbivorous beetles may indeed have contributed to the success of their plant-feeding adaptation, making some of them into notorious agricultural pests. However, a major drawback of this transcriptome sequencing analysis [27] lies in the absence of a connection between the presence of transcripts encoding putative PCWDEs in phytophagous beetles and their physiological functions. In other words, we still do not know if these genes encode functional proteins able to degrade plant cell wall polysaccharides. To address this problem, we decided to take a different approach, by first identifying beetle-derived enzymes responsible for the degradation of plant cell wall polysaccharides using a proteomics approach and then comparing them to the total number of putative PCWDE-encoding transcripts found in the corresponding transcriptome.

Here, we extend a study by Girard & Jouanin [21], by assaying the proteome of larval *Phaedon cochleariae* (Mustard leaf beetle) gut contents for degrading activities towards four plant cell wall-derived polysaccharides: cellulose, xylan, mannan and pectin. To identify the enzymes responsible for these degrading activities, we used a two-dimensional proteomics approach, first separating gut content proteins by anion exchange chromatography followed by high-resolution SDS-PAGE of protein-containing fractions. Combining this approach with either diffusion plate assays or zymograms helped us define which protein bands to further analyze by mass spectrometry. Protein identification was achieved by searching MS spectra obtained from tryptic-digested peptides against diverse protein databases, including a *P. cochleariae* transcriptome database generated by combining sequencing information obtained from Sanger and both 454 and Illumina NextGen sequencing technologies. We found that several host plant-derived proteins are very stable and persist undegraded in the insect digestive tract. We also showed that there was a discrepancy between the number of PCWDEs identified by proteomics analyses and the total number of putative PCWDE-encoding transcripts found in the transcriptome. This discrepancy was partially explained by the relative abundance of PCWDE-encoding transcripts in *P. cochleariae* midguts, an abundance that we determined by quantitative PCR and RNA-SEQ experiments.

## Results and discussion

### Degradation of plant cell wall polysaccharides by *P. cochleariae* larval gut contents

**Figure 1:**
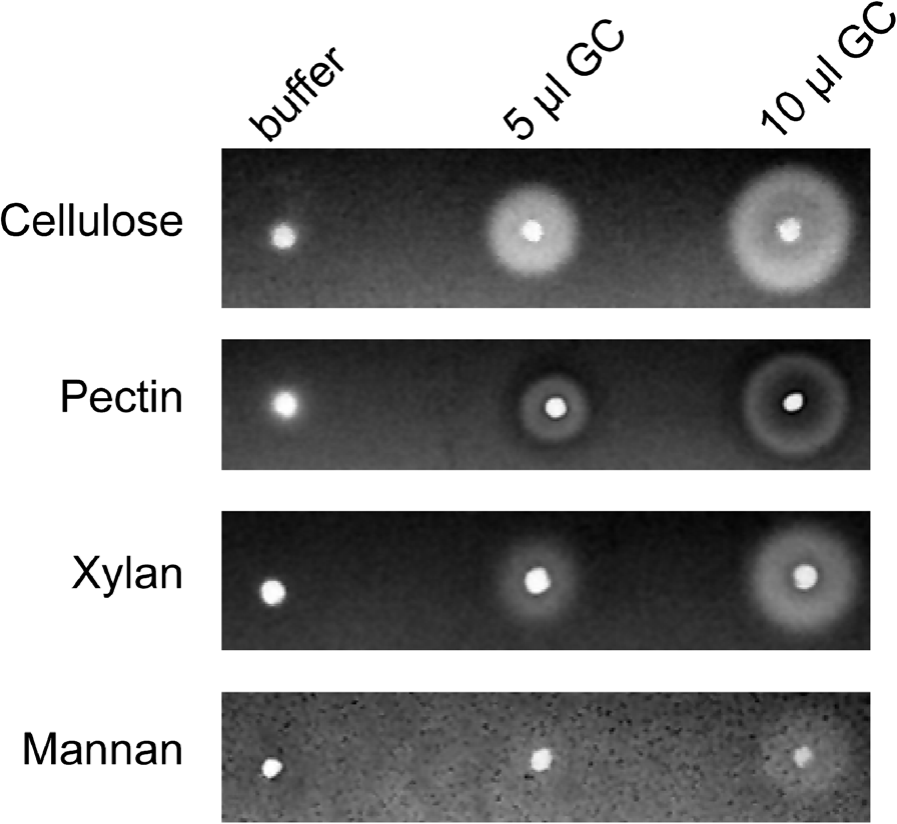
**Gut contents of *P. cochleariae *larvae degrade several plant cell wall polysaccharides.** Gut contents (GC) prepared from third-instar *P. cochleariae* larvae were tested for activity against carboxymethylcellulose, pectin from citrus peels, beechwood xylan and galactomannan by agarose plate diffusion assays. Five and 10 μl GC were deposited for each substrate as well as buffer only as control. After incubation for 1 h at 30°C, enzymatic activities (clearing zones on a dark background) were revealed by staining either with Congo Red or with Ruthenium red (pectin).

Enzymatic activities against carboxymethylcellulose (CMC), xylan and pectin have already been described for whole gut extracts from *P. cochleariae* larvae [21]. Nevertheless, we decided to repeat these experiments using gut contents rather than whole gut homogenates. In beetles, enzymes thought to be responsible for the degradation of plant cell wall polysaccharides are likely to be secreted because of the presence of a predicted signal peptide at the amino-terminus of their respective amino acid sequences [12,14,15,21-25,27,28]. Therefore, these proteins should be enriched in gut contents which, in insects, contain mainly digestive enzymes secreted by midgut cells in direct contact with the food bolus [29]. Indeed, in diffusion assays, the gut contents from *P. cochleariae* larvae exert strong enzymatic activity against CMC, pectin and xylan, but no activity against galactomannan could be detected (Figure 1). These findings correlate well with what has been previously observed in this coleopteran species [21].

**Figure 2:**
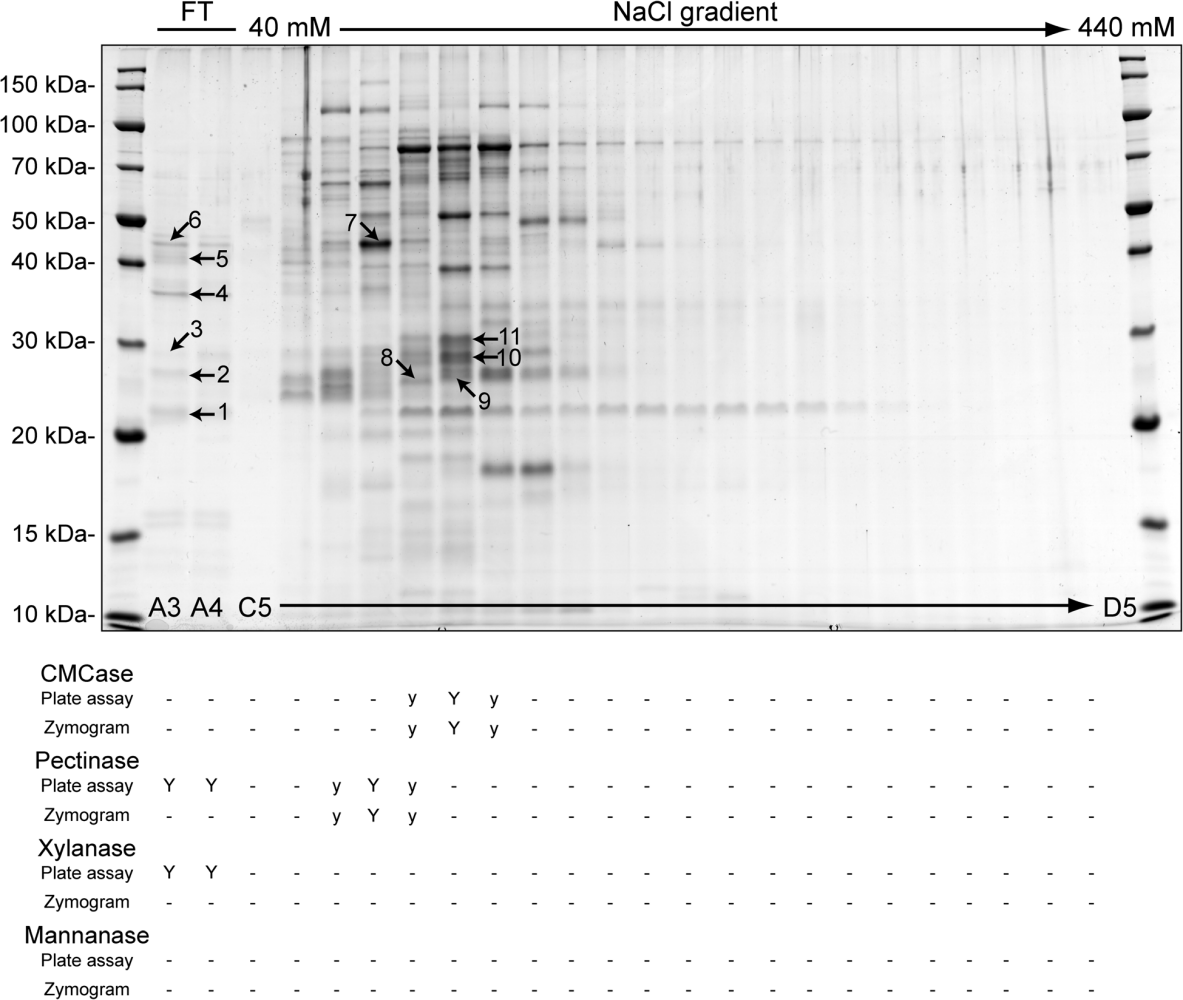
**Separation of proteins from *P. cochleariae *larval gut contents using a two-dimensional proteomics approach.** Gut content proteins were separated by anion exchange chromatography (first dimension) and 12.5% SDS-PAGE (second dimension) followed by staining with colloidal Coomassie blue. Molecular weight markers in kilodaltons are indicated to the left of the gel. Proteins that bound to the anion exchange column were eluted between 40 and 440 mM NaCl. FT: Flowthrough. Fraction numbers from the anion exchange chromatography are indicated at the bottom of the gel (see chromatogram on Figure S1). Protein bands analyzed by mass spectrometry are designated by numbers. Enzymatic activities observed for each fraction by both diffusion assays and zymograms are indicated. ‘Y’ indicates strong activity and ‘y’ low activity.

In 1999, Girard & Jouanin [21] demonstrated that *P. cochleariae* larval midgut homogenates were capable of degrading cellulose, pectin and xylan, and that transcripts encoding putative PCWDEs were actually present in a *P. cochleariae* gut cDNA library. Nonetheless, these scientists did not show that the transcripts they sequenced from the cDNA library encoded the proteins responsible for the enzymatic activities they described. To address this, we combined a proteomics approach to transcriptome sequencing to identify the proteins responsible for these enzymatic activities. Initially, we separated gut content proteins by anion exchange chromatography similar to our previous work [30], considering this step as the ‘first dimension’ of a two-dimensional approach. A large portion of these proteins bound to the column and were eluted with NaCl concentration ranging from 40 to 440 mM. After resolving these fractions by 1D/SDS-PAGE, which corresponds to the ‘second dimension’ of our two-dimensional approach, we used Coomassie blue staining to reveal a relatively simple pattern of distinct protein bands (Figure 2 and Additional file 1: Figure S1). Each fraction was tested for enzymatic activity towards CMC, xylan, pectin and mannan by two independent methods: (i) diffusion assays on agarose plates and (ii) zymograms using SDS-PAGE in ‘semi native’ conditions. CMCase activity was very intense according to both diffusion assay and zymogram (Additional file 1: Figure S2A) and was detected in the same fractions with both techniques (Figure 2). Guided by the pattern obtained using zymogram (Additional file 1: Figure S2A), we decided to pick protein bands number 8, 9, 10 and 11. Strong xylanase activity could be detected only in fractions from the flowthrough (Figure 1); no clear activity pattern could be obtained by zymogram (data not shown). Similarly, pectinase activity was detected in the flowthrough by diffusion assay but not by zymogram. We decided therefore to pick all visible protein bands from the flowthrough fraction with an apparent molecular weight above 20 kDa for tentative identification by mass spectrometry (protein bands 1 to 6). Pectinase activity was also detected in bound fractions from the anion exchange chromatography both by diffusion assay and by zymogram (Additional file 1: Figure S2B), which led us to pick protein band 7. The activity patterns we obtained by zymogram using CMC and pectin as substrates are very similar to those obtained by Girard & Jouanin [21]. Altogether 11 protein bands were collected and analyzed by mass spectrometry for tentative identification.

### Protein identification strategy

We decided to perform and compare two independent mass spectrometry analyses: (i) a ‘classical’ LC-MS/MS data-dependent acquisition method (DDA), and (ii) a relatively recent LC-MS^E^ data-independent acquisition method (DIA) [31]. Importantly, the second method has been shown to improve protein and proteome coverage compared to the conventional LC-MS/MS approach [32]. In general, protein identifications obtained by both methods were in accordance, with the better coverage generally observed with LC-MS^E^ compared to LC-MS/MS (Tables 1 and 2). Identifications were only obtained either by LC-MS/MS in four cases or by LC-MS^E^ in five cases (Tables 1 and 2). In addition, data obtained from the LC-MS/MS analyses were searched against various databases using Mascot and were alternatively interpreted *de novo* and searched against the same databases using MS BLAST [33]. We performed the MS BLAST searches to confirm and increase the confidence of the Mascot searches. We found that all MS BLAST searches were in accordance to the identifications obtained using Mascot except in the case of protein band 11 for which we found substantial differences between both search methods (see below). Altogether, this strategy resulted in highly confident protein identifications with a very limited rate of false positives. Detailed information regarding protein identifications is given as supplementary information (Additional file 2).

**Table 1:** **Summary of the identifications obtained for plant proteins using both LC-MS/MS and LC-MS**^**E**^**analyses**

**Protein band**		**Protein identification**			**Mascot (LC-MS/MS)**			**PLGS (LC-MS**^**E**^**)**		
**nb**	**MW**	**Description**	**Accession**	**MW**	**Score**	**Peptides**	**Coverage**	**Score**	**Peptides**	**Coverage**
1	22.3	*B. rapa* class IV Chitinase	BAH03380	28.7	115	2	13	2218	3	19.8
		*B. rapa* thaumatin-like	AAN23104	9.2*	-	-	-	5885	5	42.8
2	26.3	ni								
3	28.6	ni								
4	35.6	*B. juncea* β-1,3-glucanase	ABC94638	37.8	574	8	32	10332	43	47.2
		*B. rapa* β-1,3-glucanase	BAG68207	40.3	207	3	10	491	5	10.2
5	41.1	*B. rapa* Polygalacturonase-inhibiting prot 3	ACP28176	37.6	94	4	14	-	-	-
		*B. napus* peroxidase	CBD08867	38.4	-	-	-	1878	21	14.6
6	44.3	*A. lyrata* peroxidase 33	XP_002875944	39.0	190	4	9	-	-	-
		*B. napus* peroxidase	CAW62882	38.9	-	-	-	1617	27	13.8
7	43.7	*B. rapa* Polygalacturonase-inhibiting prot 3	ACP28176	37.6	165	5	18	-	-	-
8	25.3	ni								
9	25.6	*A. lyrata* glycosyl hydrolase family 38	XP_002871594	116.3	133	3	2	561	11	3.6
10	28.1	*B. napus* cysteine protease	ABA71355	39.4	-	-	-	2039	11	15.3
11	30.1	ni								

*This accession corresponds to a partial protein sequence. ni: no identification.

**Table 2:** **Summary of the identifications obtained for *Phaedon cochleariae*-derived proteins using both LC-MS/MS and LC-MS**^**E**^**analyses**

**Protein band**		**Protein identification**			**Mascot (LC-MS/MS)**			**PLGS (LC-MS**^**E**^**)**		
**nb**	**MW**	**Description**	**Accession**	**MW**	**Score**	**Peptides**	**Coverage**	**Score**	**Peptides**	**Coverage**
1	22.3	ni								
2	26.3	GH11-1	HE962209	23.9	155	2	15	2642	4	24.8
		GH11-2	HE962210	23.7	116	4	22	1687	13	24.8
3	28.6	ni								
4	35.6	GH28-7	HE962199	40.9	117	5	11	1425	5	11.8
		3-hydroxyacyl-CoA dehydrogenase	Contig11024	36.1	191	3	10	835	9	14.3
5	41.1	GH28-9	HE962201	38.2	261	5	18	2188	16	28.1
		GH28-6	HE962198	40.7	144	3	10	1172	5	15.5
6	44.3	ni								
7	43.7	GH28-1	HE962193	38.8	185	5	14	6650	54	29.2
		GH28-3	HE962195	39.8	119	3	11	-	-	-
		GH16-1	Contig27637	42.7	197	5	13	1904	16	22.2
		GH1-1	Contig25268	33.2*	269	6	22	3028	17	22.3
8	25.3	ni							-	
9	25.6	Cysteine proteinase	-	20.9*	-	-	-	6518	11	54.8
10	28.1	GH45-4	HE962204	25.6	76	1	6	5377	9	30.4
		GH45-5	HE962205	25.4	99	2	13	1183	5	29.2
11	30.1	GH45-7	HE962207	25.8	-	-	-	2314	12	9.9

*This accession corresponds to a partial protein sequence. ni: no identification.

### Stability of host plant-derived proteins in *P. cochleariae* gut contents

As the insects we used were fed on Chinese cabbage plants and not on artificial diet, we hypothesized that host plant-derived proteins could also be present in *P. cochleariae* gut contents. To evaluate the presence of Chinese cabbage-derived proteins in our sample, we subjected the data we obtained from both LC-MS/MS and LC-MS^E^ analyses to a search against a Viridiplantae protein database. Several proteins derived from plants in the Brassicaceae, including Chinese cabbage, were confidently identified (Table 1 and Additional file 2). Many of these proteins are common in plants and belong to several families of pathogenesis-related proteins (PRs). Among these, a β-1,3-glucanase (PR-2), a type IV chitinase (PR-3), a thaumatin-like protein (PR-5) and a peroxidase (PR-9) were indentified (Table 1). The MWs observed on the gel of the protein bands from which PRs were identified are similar to the MWs of the predicted proteins, indicating that these plant-derived proteins seem to remain intact in the insect gut lumen. PRs are defense-related proteins inducible upon infection with phytopathogenic fungi or bacteria, as well as viruses and even insect attack [34]. Most PRs can be induced through the action of signaling compounds such as salicylic acid, jasmonic acid, or ethylene and were shown to exhibit antimicrobial activities through either the ability to hydrolyze cell walls or contact toxicity, and may also be involved in defense signaling [34]. The full-length protein sequences of all the PRs we identified here possess an amino-terminus signal peptide, indicating that their location in plants is the intercellular space. Their compact structure, often stabilized by disulfide bridges, makes PRs extremely tough proteins. Resistant towards proteolysis and elevated temperature, PRs remain soluble at low pH, allowing them to survive in harsh environments, including the gut lumen of insect herbivores [35].

Protein bands 5 and 7 contain peptides corresponding to polygalacturonase-inhibiting proteins (PGIPs) from *Brassica napus* (Table 1). PGIPs are glycoproteins associated with the plant cell wall which are believed to play an important role in defense against phytopathogenic fungi. Their main function is to target fungal-derived polygalacturonases and reduce their hydrolytic activity towards plant cell wall pectins, resulting in a negative effect on fungal growth [36]. The typical primary structure of PGIPs comprises an amino-terminal signal peptide for secretion and a mature polypeptide characteristic of proteins from the leucine-rich repeat (LRR) superfamily [37]. Although PGIPs are not classified as PRs, their expression can also be induced by both biotic (phytopathogenic fungi and insects) and abiotic (wounding, phytohormones) elicitors, and PGIPs play an active role in plant defense [38]. Similar to PRs, the protein bands containing peptides corresponding to PGIPs have MWs close to the ones predicted from protein sequences (Table 1), indicating that these PGIPs seem to be resistant to proteolysis by insect-derived digestive proteinases. The apparent stability of both PRs and PGIPs in *P. cochleariae* gut contents together with what is known about their physiological functions indicates that both protein families are potential candidates for plant defense against this herbivorous insect.

### Identification of PCWDEs from *P. cochleariae* gut contents

To specifically identify insect-derived proteins from the 11 protein bands we analyzed, we searched the resulting mass spectrometry data against a *P. cochleariae*-derived protein database established from transcriptome data generated in-house, and translated *in silico* in the six possible open reading frames. To improve the significance of our identifications, we merged this database to the Swiss-Prot protein database. From the 11 protein bands analyzed, we positively identified 17 insect-derived proteins (Tables 2 and 3). Out of these, 13 correspond to putative PCWDEs according to our previous study [27] and are classified in 3 glycoside hydrolase (GH) families based on the CAZy nomenclature [39], GH11, GH28 and GH45.

**Table 3:** **Identification of *Phaedon cochleariae*-derived proteins for protein band 11 using sequence-similarity based searching (MS BLAST) of peptides obtained by *de novo *sequencing from LC-MS/MS data**

**Protein identification**			**MS BLAST results**		
**Description**	**Accession**	**MW**	**Score**	**Peptides**	**Coverage**
GH45-1	HE962202	25.5	471	7	35.5
GH45-7	HE962207	25.8	344	5	23.2
GH45-3	HE962203	25.6	308	5	23.9
GH45-4	HE962204	25.6	301	5	23.3

Xylanase activity was restricted to the flowthrough, therefore proteins corresponding to putative xylan-degrading enzymes should be present in one of the corresponding protein bands we analyzed (bands 1 to 6). Proteins known to exhibit xylanase activity are restricted to four GH families (GH5, GH8, GH10 and GH11) according to CAZy [39], and protein hits for one or more of these enzyme families were expected. Indeed, peptides from protein band 2 hit two distinct proteins possessing a GH11-conserved domain (Table 2). One of them, GH11-1, corresponds to a transcript previously identified in *P. cochleariae* (CAA76932.1) [21], whereas GH11-2 is new to this study. These two proteins share almost 80% amino acid identity, both harbor a 17 amino acid signal peptide at their amino-terminus, and the two predicted catalytic residues (glutamate) are conserved, indicating that both proteins are potentially active enzymes (Additional file 1: Figure S3). No hit for one of the other three GH families was obtained, suggesting that these two GH11 enzymes represent our sole candidates for the xylanase activity observed.

Pectinase activity was also detected in the flowthrough, but only by diffusion assays and not by zymograms (Figure 2). Known pectin-degrading enzymes, or polygalacturonases, are members of a single GH family (GH28) according to CAZy, and peptides corresponding to protein bands 4 and 5 gave hits for three distinct proteins with a conserved GH28 domain (GH28-7 in protein band 5; GH28-6 and −9 in protein band 6). Our diffusion assays (Figure 1) and the zymogram (Additional file 1: Figure S2B) also indicated that peptides from protein band 7 should also hit polygalacturonases, and indeed we identified two additional GH28s (GH28-1 and −3). These five GH28s all harbor a signal peptide at their amino-terminus and conserved putative catalytic residues, except for GH28-3 for which the third putative catalytic aspartate residue is substituted by an asparagine, suggesting that GH28-3 may not be an active enzyme (Additional file 1: Figure S4). Taking this into account, the polygalacturonase activity detected by zymogram corresponding to protein band 7 (Additional file 1: Figure S4) may be due only to GH28-1, whereas the activity detected from the flowthrough may represent the common contribution of GH28-6, -7 and −9. Notably, GH28-1 corresponds to a polygalacturonase transcript (Y17906.1) from Girard & Jouanin [21], except for the presence of four frameshifts compared to the previously described sequence. We believe that our sequence is correct based on the high number of clones we sequenced, and also because the corresponding protein shares 83% amino acid identity with its closest relative in the other chrysomelid *Chrysomela tremula* (ADU33280.1), whereas the protein corresponding to the previously described sequence shares only 79% with the same accession from *C. tremula*.

CMCase activity was detected by both diffusion plate assays and zymograms, corresponding to proteins that are bound to the anion exchange chromatography column, centered on the fraction containing protein bands 9, 10 and 11 (Figures 2 and Additional file 1: Figure S2A). Peptides corresponding to protein bands 10 and 11 hit five proteins harboring a GH45 conserved domain present in the *P. cochleariae* protein database (Tables 2 and 3), namely GH45-4 and −5 from protein band 10 and GH45-1, -7 and −3 from protein band 11. Similar to GH11s and GH28s, these proteins possess a signal peptide, and their catalytic residues (two aspartates) are conserved (Additional file 1: Figure S5). The conservation between GH45-4 and −5 is very high; both sequences share 88.4% amino acid identity, which was also reflected in the MS data we obtained from protein band 10. In fact the LC-MS^E^ analysis revealed that in several cases, the same peptide from this protein band matches both sequences; however, four peptides were found to be ‘discriminating’, meaning that they correspond to the same region on both GH45-4 and −5 where amino acid variations occur. For example, peptide QLLVQVTNTGSDLGK (m/z 1572.865) matches GH45-4, whereas peptide QMLVQVTNTGSDLGK (m/z 1606.8145) matches GH45-5. Similarly, peptide YGGVHTEEECNQLPEDLQEGCK (m/z 2592.119) matches GH45-4, whereas peptide ITGVQTEEECNQLPEDLQEGCK (m/z 2577.1619) matches GH45-5. Therefore, we can conclude with certainty that both GH45-4 and −5 are present in protein band 10, and that matches to one or the other do not represent a false positive identification (Additional file 2). Again, notably, GH45-1 corresponds to a previously described sequence (Y17907.1) [21], except for the presence of four frameshifts. Our sequence shares 69% amino acid identity with its closest relative in *Leptinotarsa decemlineata* (ADU33350.1), whereas the previously described sequence shares only 64% with the same sequence in *L. decemlineata*. Taking this into account together with the number of clones we sequenced, we believe that our sequence is correct.

### Identification of insect-derived proteins other than PCWDEs

Together with the 13 protein identifications for PCWDEs, we obtained four hits for other insect-derived proteins (Table 2). Among these, two proteins from other GH families were identified: one with a GH16 conserved domain and the other one with a GH1 conserved domain. GH16-1 is very similar to lepidopteran and termite-derived β-1,3-glucanases for which the function is still controversial. In fact, these enzymes have been implicated in the digestion of β-1,3-glucans found in fungal cell walls and been found to be part of the immune system of Lepidoptera and termites [40-42]. Peptides from protein band 7 matched to a partial sequence corresponding to a putative β-glucosidase (GH1-1). Aside from the obvious role of GH1 proteins in digestion [43], a member of this family in the cabbage aphid, *Brevicoryne brassicae*, has been functionally characterized as a myrosinase [44,45]. Myrosinases have been extensively studied in plants from the family Brassicaceae because these enzymes mediate the hydrolysis of glucosinolates, the main secondary metabolites found in these plants, leading to a labile aglycone, which rapidly undergoes spontaneous rearrangement to yield a variety of toxic metabolites such as isothiocyanates, thiocyanates, cyanoepithioalkanes and nitriles. Plant myrosinases and glucosinolates constitute a system in cruciferous plants that defends against pests (insects and phytopathogenic microorganisms) and diseases. Insect-derived myrosinase may play a role that is similar to the role it plays in plants, protecting crucifer-feeding insects against predation by making them distasteful, due to the hydrolysis of glucosinolates present in their diet [44]. *Phaedon cochleariae* feeds exclusively on plants from the family Brassicaceae, and although the presence of a myrosinase activity has not been yet identified in this species, the hypothesis that such enzyme is present cannot be excluded.

Additionally, two other non-PCWDE proteins have been identified: one, a cysteine proteinase, and the other, a putative 3-hydroxyacyl-CoA dehydrogenase. Beetles from the infraorder Cucujiformia rely mainly on cathepsin-type cysteine proteinases to digest proteins, with little contribution from serine proteinases (trypsins and chymotrypsins) [43]; therefore their presence in *P. cochleariae* is to be expected. The presence of other cathepsins in protein bands which were not examined here is also to be expected. The presence in *P. cochleariae* gut contents of a putative 3-hydroxyacyl-CoA dehydrogenase, an enzyme implicated in lipid metabolism in peroxisomes, is difficult to understand. The substrate for this enzyme is expected to occur only intracellularly. The predicted protein does not harbor a signal peptide at its amino-terminus and therefore should not be secreted by the canonical pathway. We presume that it may be released into the lumen by occasional cell breakage or as an "accidental" passenger in an exocytotic pathway, and that persists in the lumen due to resistance to proteases. An analogous example from Lepidoptera is arginine kinase, found in the gut lumen of species such as *Helicoverpa armigera*[29] and *Plodia interpunctella*[46] where it has been shown to be a human allergen.

### Co-isolation of *P. cochleariae* PCWDEs and host-plant-derived proteins

A very interesting observation from our proteomic analyses is the finding of both insect-derived PGs and plant-derived PGIPs in the same fraction from the anion exchange chromatography and even the same protein band (Tables 1 and 2). Their presence in the same protein bands in the gel (5 and 7) could be attributed to their highly similar molecular weight. On the other hand, it is unlikely that their physico-chemical properties are so similar that they show the same anion exchange behavior; thus their occurrence in exactly the same fractions from the anion exchange chromatography suggests a binding interaction *in vivo*. PGIPs occur as a multigene family in plants, two members are found in the model plant *Arabidopsis thaliana*[47] and up to nine members in the recently sequenced genome of *Brassica rapa*[48]. These proteins have been extensively studied for their role in defense against fungal pathogens [4], but their ability to inhibit insect-derived polygalacturonase activity has also been described. For example, polygalacturonase activity from two mirid bugs, *Lygus rugulipennis* and *Adelphocoris lineolatus*, is strongly inhibited by two PGIPs (PGIP3 and PGIP4) from the common bean *Phaseolus vulgaris*[49,50]. Similarly, an endopolygalacturonase purified from the sugarcane rootstalk borer weevil, *Diaprepes abbreviatus*, is inhibited in a concentration-dependent manner by a semi-purified PGIP from orange flavedo [51]. Altogether, this indicates that PGIPs from Chinese cabbage (*B. rapa* ssp. *pekinensis*), the plant we used to feed *P. cochleariae*, may play an active role in defense against this insect by inhibiting one or several of its polygalacturonases. At this stage of our analysis, we do not know which of the PGs (GH28-6, -7 or −9) binds the PGIP protein we identified from the flowthrough. The same applies to the PGIP identified in the same fraction from the anion exchange chromatography as GH28-1 and −3. To address this uncertainty, experiments testing the direct binding of PGIPs to individual *P. cochleariae*-derived PGs need to be performed. What we can note, however, is that *P. cochleariae* may have partially overcome this line of plant defense, as polygalacturonase activity can still be detected in its gut contents (Figure 1 and Additional file 1: Figure S2B.) after feeding on *B. rapa*, which may contribute to adaptation to one of its host plants.

### Survey of the *P. cochleariae* transcriptome for putative PCWDE transcripts

Using sequence similarities and BLAST searches, we identified 19 transcripts encoding putative PCWDEs in the *P. cochleariae* transcriptome we generated from pooled mRNA of all larval and adult tissues. Two encode putative xylanases (GH11), nine, putative polygalacturonases (GH28), and eight, putative cellulases (GH45). One, named GH45-2, is most likely a pseudogene, as the open reading frame is interrupted by a premature stop codon. All the other 18 transcripts potentially encode putatively secreted functional proteins, as they all harbor an amino-terminal signal peptide. This is in contrast to the number of identifications we obtained from the proteomic analysis. Although the proteins corresponding to the two xylanase transcripts were identified, only five out of nine GH28 and five out of seven GH45s could be identified. Three hypotheses could account for these observed discrepancies between the number of proteins identified based on their enzymatic activity and the number of putative transcripts from the transcriptome. First, some of these transcripts could be expressed in tissues other than the insect gut. Second, the expression of some of these transcripts may be very low in ‘normal’ feeding conditions, for example, when the insect feeds on a plant to which it is highly adapted. Third, the proteins may be present in gut contents but were not identified because they do not degrade the substrates that we tested.

**Figure 3:**
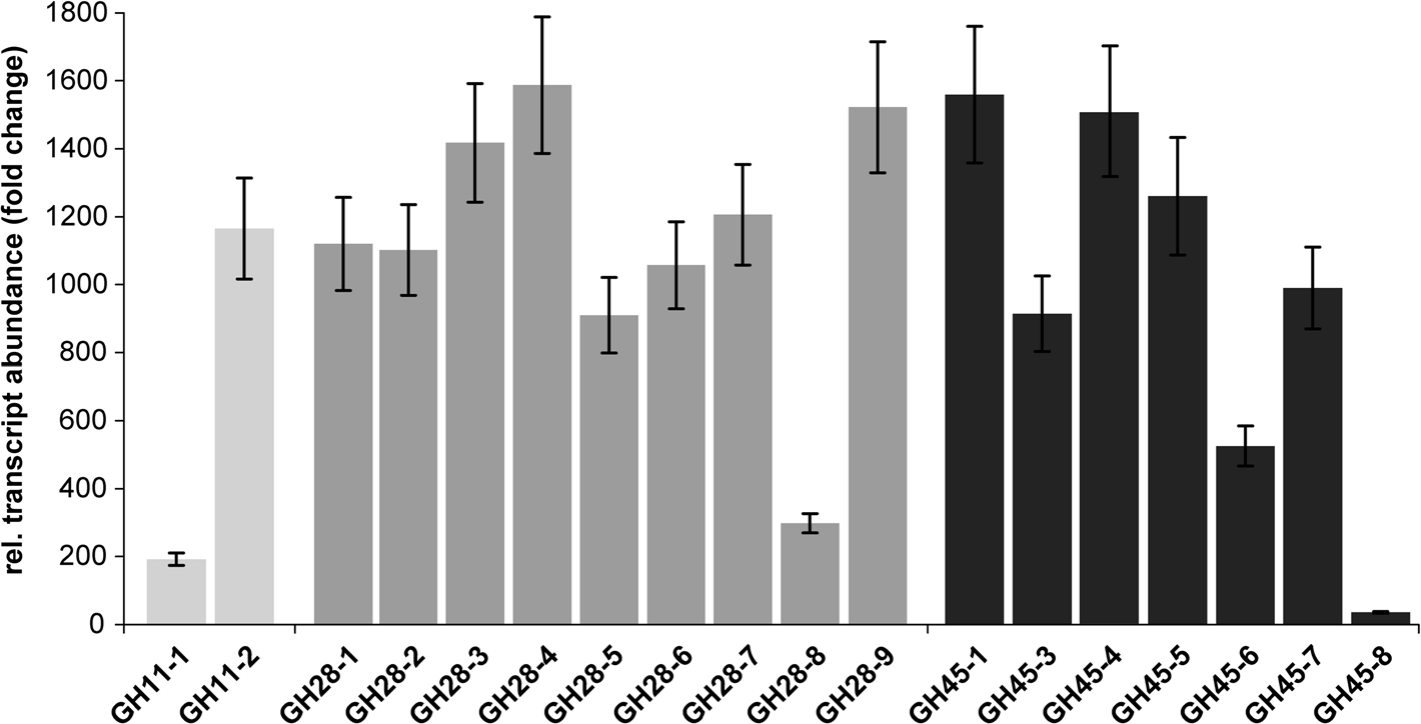
**Relative abundance of transcripts encoding GH11 (light gray), GH28 (gray) and GH45 (dark gray) proteins in *P*. *cochleariae *larvae.** All PCWDE-encoding genes are specifically expressed in the gut and 20 to 1600 fold up-regulated compared to the rest of the body. For normalization of transcript quantities, EF1α and eIF4A were used as reference genes. Fold changes were calculated by setting relative transcript abundances to a value of 1 for the rest body. Error bars represent the SEM.

To evaluate these three possibilities, we first performed quantitative real-time PCR experiments in which we compared the expression of these 18 transcripts in the gut tissue compared to the expression of transcripts in the rest of the insect body (Figure 3). Without exception, all putative PCWDE transcripts are specifically expressed in the gut compared to the rest of the body; therefore, we rejected the first hypothesis. For insight into the expression levels of each individual putative PCWDE-encoding transcript, we mapped all RNA-SEQ reads of the *P. cochleariae* transcriptome to these transcripts using a mapping and quantification tool (Table 4). These reads came from four ‘pools’, larval gut and rest body, as well as adult gut and rest body. This analysis clearly showed that all transcripts are predominantly expressed in the insect gut rather than the rest of the body, confirming the results we obtained from quantitative real-time PCR experiments. Moreover, these data show that there is almost no difference in the expression of these genes in larvae and in adults, which we also hypothesized as both developmental stages have the same feeding regimen (Table 4). Likewise, proteins corresponding to GH28 and GH45-encoding transcripts with the highest expression were identified in our proteomics approach, the top three for GH28s and the top two for GH45. In contrast, the proteins corresponding to the two GH28 transcripts and one GH45 transcript displaying the lowest expression in the RNA-SEQ analysis could not be identified in our proteomics approach. These low mRNA expression levels are most likely also reflected at the protein level. The expression of both GH28-8 and −5 is about 50 times lower than that of GH28-9, the most highly expressed GH28. Similarly, the expression of GH45-6 is about 70 times lower than that of GH45-1. However, in contrast, the apparent absence from the gut content proteome of GH28-2 and −4, as well as of GH45-8 cannot be correlated with the expression level of their corresponding transcripts, which partially invalidates our second hypothesis. Finally, we had a closer look at the amino acid sequences of all putative PCWDEs. In all cases except one, GH28-3, putative catalytic residues were detected (Additional file 1: Figure S3, Additional file 1: Figure S4 and Additional file 1: Figure S5). As noted above, a substitution of a catalytic aspartate residue to an asparagine occurred in GH28-3; nonetheless, the corresponding protein could be identified from protein band 7, indicating that although GH28-3 is most likely not an active polygalacturonase, it may have evolved another function in *P. cochleariae*. The only way for us to determine whether a given enzyme possesses the proposed activity is by functional characterization, which represents one of our future objectives.

**Table 4:** **Transcript abundance of each GH11, GH28 and GH45 in *P*. *cochleariae *larvae and adults based on a mapping of Illumina reads to the ORFs of the PCWDEs**

**Name**	**cds (bp)**^***a***^	**LG**^***b***^		**LR**^***b***^		**AG**^***b***^		**AR**^***b***^	
	**Reads**^***c***^		**RPKM**^***d***^	**reads**^***c***^	**RPKM**^***d***^	**reads**^***c***^	**RPKM**^***d***^	**reads**^***c***^	**RPKM**^***d***^
**GH11**									
GH11-2*	654	7091	14,46935	18	7,59882	4098	13,81739	2	4,09316
GH11-1*	654	4018	13,64994	13	7,12934	2375	13,03126	1	3,09316
**GH28**									
GH28-9*	1113	104975	17,62099	420	11,3895	108541	17,8029	69	8,51589
GH28-1*	1101	52323	16,60188	182	10,18524	33084	16,07982	16	6,3417
GH28-3*	1116	25617	15,55142	121	9,58866	23003	15,53571	39	7,60758
GH28-4	1110	17208	14,98799	42	8,05802	14477	14,87829	54	8,08485
GH28-2	1089	10944	14,36062	64	8,69326	12377	14,67758	31	7,31171
GH28-7*	1125	10075	14,19475	33	7,69073	20121	15,33691	19	6,55852
GH28-6*	1128	5395	13,28797	18	6,81242	8822	14,13731	10	5,62868
GH28-8	1125	2254	12,03587	18	6,81626	2093	12,07132	14	6,11795
GH28-5	1110	2213	12,02813	30	7,5758	2618	12,40896	23	6,85352
**GH45**									
GH45-1*	729	25102	16,13723	104	9,97271	21358	16,04375	15	6,84342
GH45-3*	732	15902	15,51018	102	9,96678	13460	15,39577	33	8,05989
GH45-8	729	15813	15,47135	73	9,46209	17763	15,77897	21	7,32885
GH45-7*	726	13114	15,20774	60	9,18511	15761	15,61229	24	7,52744
GH45-4*	723	6088	14,11546	21	7,67651	10930	15,09838	8	6,11838
GH45-5*	723	4651	13,72137	27	8,03908	6289	14,2971	22	7,40788
GH45-6	726	365	10,04911	1	3,27822	557	10,79833	12	6,52744
**References**									
eIF4A	1263	11457	14,21229	14111	16,26465	10960	14,28746	11633	15,64997
EF1α	1389	67267	16,62849	90280	18,80497	63998	16,69593	61492	17,91526

^*a*^Length of the full length coding DNA sequences (cds) in base pairs. ^*b*^Sequences were generated from four independent RNA pools: larval gut (LG), larval rest body (LR), adult gut (AG) and adult rest body (AR). ^*c*^Absolute numbers of reads mapped to each ORF corresponding to a putative PCWDE for each specific RNA pool. ^*d*^The number of reads per kilobase and per million (RPKM values) are log_2_ transformed (see ‘Experimental section’). * indicates that the corresponding proteins were identified from the proteomic analysis.

We clearly demonstrated that transcripts encoding putative PCWDEs are actively expressed in the gut tissue, suggesting that the corresponding proteins should be present in gut contents. However, we cannot yet exclude the possibility that the respective proteins get trapped after secretion, either in the glycocalyx present at the surface of gut cells or in the peritrophic matrix before they could reach the gut lumen [43,52,53]. The peritrophic matrix is a hollow meshwork tube of chitinous fibres cross-linked by proteins which have several well-described functions in insects, such as protecting the gut epithelium against physical damages or infection and compartmentalizing digestive processes, and trapping digestive enzymes and other proteins [52,53]. Further proteome analyses targeting these two compartments of the insect gut need to be performed to address thisx issue.

Finally, we also cannot exclude the possibility that the PCWDEs that were not identified after the proteomics approach could indeed be present in the 11 protein bands we analyzed by mass spectrometry; but that technical limitation of mass spectrometry itself prevented their identification. In fact, protein identification by mass spectrometry is conditioned by the fact that tryptic peptides must be ionized to be fully analyzed. In other words, only peptides that were properly ionized are considered for database searches. In addition, although we already considered some peptide modifications for our database searches, such as carbamidation of cysteines or oxidation of methionines, other types of modifications can occur and impair database searches and protein identifications. Among these, N-linked glycosylation, which is a common feature of secreted proteins and of the putative PCWDEs we identified (Table S1), can dramatically change the apparent ion masses of tryptic peptides and prevent protein identification.

## Conclusions

We have demonstrated that combining transcriptomics and proteomics represents a powerful approach for protein discovery and enables confident identifications to be made in non-model insects, such as the mustard leaf beetle *P. cochleariae*. We found that several putative PCWDE genes found in phytophagous beetles encode functional enzymes. It is now clear that the overall degradation of plant cell wall polysaccharides, at least in *P. cochleariae*, is due to multiple members of several gene families of insect-derived enzymes rather than single members of several gene families, as previously thought [21]. Although transcriptome sequencing by itself represents a very efficient method for gene discovery, we believe that attributing a function and/or annotating a gene based only on sequence similarities, without performing any kind of functional characterization, is generally insufficient and may lead to a false interpretation of the physiological role of a given protein or protein family. Consequently, our next task will consist of functionally characterizing every single putative PCWDE we identified here. Finally, our data indicated that, although the insect digestive system is very efficient in digesting plant material, some host plant-derived proteins remain stable and resist proteolysis. The identification and characterization of these highly resistant plant proteins, as well as their potential targets within the insect digestive system, may provide crucial information on key aspects of the ‘arms race’ between the insect and its host plant.

## Methods

### Insect and plant rearing

*Phaedon cochleariae* was collected on Brassicaceae close to the city of Bayreuth (Germany). Larvae and adults were kept as a continuous culture in the laboratory, at 20°C and on a cycle of 16 h light/ 8 h dark, on leaves of Chinese cabbage (*Brassica rapa* sp. *pekinensis*). Chinese and white cabbage (*Brassica oleracea* convar. *capitata* var. *alba*) plants were reared in the greenhouse (21°C, 55% humidity, long day conditions, 14 h/10 h light/dark period).

### Protein extraction and gel electrophoresis

Twenty-five third-instar larvae were dissected in 100 mM citrate/phosphate buffer pH 5.0 containing a cocktail of protease inhibitors (Complete EDTA-free, Roche). Intact whole guts were transferred in 500 μl of the same buffer/inhibitor mixture, opened on one side and soaked in the buffer before removing them. The resulting buffer/gut content mixture was kept on ice during gut dissection and immediately centrifuged afterwards (5,000 *g*, 5 min, 4°C). The supernatant was collected and stored at −80°C until use.

The whole 500 μl gut content was loaded on a 1 ml RESOURCE Q anion exchange chromatography column (GE Healthcare) connected to an Äkta FPLC system (GE Healthcare). After extensive washing of the column, bound proteins were eluted using a 0 to 1 M linear NaCl gradient over 30 column volumes. Eluted proteins were recovered in 500 μl fractions (Additional file 1: Figure S1). One hundred microliters of each fraction containing a protein peak at 280 nm were precipitated by 10% trichloroacetic acid, using 0.02% sodium deoxycholate as a co-precipitant; the final pellets were dissolved and boiled in 10 μl SDS-PAGE sample buffer. Samples were then loaded on a Criterion 12.5% polyacrylamide SDS-PAGE gel (BioRad) and run for 2 h at a constant voltage of 120 V. Gels were then fixed in 40% (v/v) ethanol, 10% (v/v) acetic acid, for 2 h and stained using Colloidal Coomassie as described by Neuhoff et al. [54]. Finally, gels were scanned using a GS800 densitometer (BioRad) and analyzed using Quantity One® software version 4.6.3 (BioRad).

### Activity assays

To screen for relevant enzymatic activities in the gut contents of *P. cochleariae* larvae, diffusion assays were performed in 1% agarose Petri dishes containing either 0.1% carboxymethylcellulose (CMC), 0.1% beechwoodxylan, 0.1% pectic acid from citrus peels or 0.1% galactomannan and 50 mM citrate/phosphate buffer pH 5.0. Two millimeter holes were made into the agarose, and 5 μl of each fraction from the anion exchange chromatography containing a protein peak at 280 nm were added to each hole. Activity was revealed after 1 h of incubation at 30°C with 0.1% Congo Red solution (for CMC, beechwood xylan and galactomannan) or 2 h at room temperature with 0.1% Ruthenium red solution (for pectic acid); each plate was then destained with 1 M NaCl or distilled water (for ruthenium red) until pale activity zones appeared against a dark red background.

Five microliters of each fraction from the anion exchange chromatography containing a protein peak at 280 nm were prepared for zymogram by diluting them in Laemmli sample buffer without any reducing agent. Samples were run on a 12.5% SDS-PAGE gel containing either 0.1% CMC, 0.1% pectic acid from citrus peels or 0.1% beechwood xylan. Electrophoresis was carried out at 4°C using pre-chilled running buffer. Gels were then washed three times in a 2.5% Triton X-100 solution for 15 min each at 4°C before being equilibrated in reaction buffer (50 mM citrate/phosphate buffer pH 5.0) for 16 h at 4°C, followed by a 1 h incubation at 30°C. Activity was revealed as described above.

### In-gel digestion and peptide extraction

Protein bands of interest were cut out from either the zymogram gels or the Coomassie-strained gel, and tryptic digestion was carried out as described before [55]. Briefly, proteins were reduced in-gel by 10 mM dithiotreitol and alkylated by 55 mM iodoacetamide. Destained, washed, dehydrated gel pieces were rehydrated for 60 min in 0.5 μM solution of bovine trypsin in 25 mM ammonium bicarbonate buffer at 4°C and then digested overnight at 37°C. The tryptic peptides were extracted from gel pieces with extraction buffer (75% acetonitrile / 5% formic acid), and the extracts were dried out in a vacuum centrifuge. For LC-MS, analysis samples were reconstructed in 10 μL aqueous 1% formic acid.

### LC-MS/MS analysis

Samples were separated using a nanoAcquity nano UPLC system (Waters, Manchester, UK). A mobile phase of 0.1% aqueous formic acid was used to concentrate and desalt the sample on a Symmetry C18 trap-column (20 x 0.18 mm, 5 μm particle size) at a flow rate of 15 μL per min. Subsequently, peptides were eluted onto a nanoAcquity C18 column (200 mm ×75 μm ID, C18 BEH 130 material, 1.7 μm particle size) using an increasing acetonitrile gradient in 0.1% aqueous formic acid at a flow rate of 0.350 μl per min. Buffers A (0.1%FA) and B (100% MeCN in 0.1% FA) were linearly mixed in a gradient from 1% to 55% phase B over 60 min, increased to 95% B over 5 min, held at 95% B for 5 min and decreased to 1% B over 1 min. The analytical column was immediately re-equilibrated for 9 min.

The eluted peptides were transferred to the nanoelectrospray source of a Synapt HDMS tandem mass spectrometer (Waters, Manchester, UK) equipped with a metal-coated nanoelectrospray tip (Picotip, 50 × 0.36 mm, 10 μm internal diameter, New Objective, Woburn, USA). The source temperature was set to 80°C, cone gas flow 20 L/h, and the nanoelectrospray voltage was 3.2 kV. For all measurements, the mass spectrometer was operated in V-mode with a resolution power of at least 10,000 FWHM. All analyses were performed in positive ESI mode. A 650 fmol/μL human Glu-fibrinopeptide B in 0.1% formic acid/acetonitrile (1:1 v/v) was infused at a flow rate of 0.5 μL per min through the reference NanoLockSpray source every 30 seconds to compensate for mass shifts in MS and MS/MS fragmentation mode.

LC-MS data were collected using data-dependent acquisition (DDA) and data-independent LC-MS^E^ (DIA) analyses. For DDA, the acquisition cycle consisted of a survey scan covering the range of m/z 400–1500 Da followed by MS/MS fragmentation of the three most intense precursor ions collected at 1 sec intervals in the range of 50–1700 m/z. Dynamic exclusion was applied to minimize multiple fragmentations for the same precursor ions. For LC-MS^E^ analyses, full-scan LC-MS data were collected using alternating mode of acquisition: low energy (MS) and elevated energy (MS^E^) mode at 1.5 sec intervals in the range m/z of 50–1700 with a delay of 0.2 sec between scans. In low energy mode, data were collected at constant collision energy of 4 eV set on the trap T-wave device and ramped during scan from 15 to 40 eV in elevated MS^E^ mode.

### Data processing and protein identification

DDA raw files were collected using MassLynx v4.1 software and processed using ProteinLynx Global Server Browser (PLGS) v2.5 software (Waters, Manchester, UK) under baseline subtraction, smoothing, deisotoping, and lockmass-correction. Processed MS/MS spectra were searched against the NCBInr database (updated on September, 11, 2011, containing 15,270,974 sequence entries) combined with the *P. cochleariae* protein subdatabase (containing 644,940 entries, constructed from the in-house created EST database by its translation from all six reading frames; see below) using MASCOT v2.3 software installed on a local server and connected to PLGS as a search engine. Mass tolerances for precursor and fragment ions were 15 ppm and 0.03 Da, respectively. Other search parameters were as follows: instrument profile, ESI-Trap; fixed modification, carbamidomethyl (cysteine); variable modification, oxidation (methionine), deamidation (asparagine, glutamine); up to 1 missed cleavage was allowed. Hits were considered to be confident if at least three peptides were matched with ion scores above 30, or proteins were identified by one or two peptides with a total protein score of 55 or better. Ions score is -10Log(P), where P is the probability that the observed match is a random event. Individual ions scores > 25 indicate identity or extensive homology (p<0.1). Protein scores are derived from ions scores as a non-probabilistic basis for ranking protein hits.

In parallel, MS/MS spectra were searched using PLGS as a search engine against a subdatabase containing common background proteins (human keratins and trypsin) in order to exclude these spectra from *de novo* sequencing. The applied searching parameters were the same as described above. Alternatively, spectra were interpreted *de novo* to yield peptide sequences. A 0.002 Da mass deviation for *de novo* sequencing was allowed, and sequences with a ladder score (percentage of expected y- and b-ions) exceeding 40 were subjected to sequence-similarity searching using the MS BLAST program [33] installed on an in-house server. MS BLAST searches were carried out against the following subdatabases: *P. cochleariae* (containing 644,940 sequence entries), insecta subdatabase (downloaded from NCBInr on October 21, 2011, containing 1,504,915 sequence entries) and viridiplantae subdatabase (downloaded from NCBInr on October 21, 2011, containing 1,878,311 sequence entries) as well as against the complete NCBInr database (updated on August 10, 2011, containing 14,977,208 sequence entries) under following settings: scoring Table, 100; Filter, none; Expect, 100; matrix, PAM30MS; advanced options, no-gap-hspmax100-sort_by_totalscore-span1. Statistical significance of hits was evaluated according to the MS BLAST scoring scheme [33].

The continuum LC-MS^E^ data were processed by PLGS v2.5 software. Baseline-subtracted, smoothed, deisotoped, and lockmass-corrected spectra were aligned according to the ion accounting algorithm [31]. Statistics and scoring used by PLGS has been described by Skilling et al. [56]. Results are documented in the spreadsheets of Additional file 2. The processed data were searched against the following subdatabases: *P. cochleariae*, insecta and viridiplantae. The search parameters were set as follows: the same fixed and variable modifications as for searching the DDA data, one possible missed tryptic cleavage site, automatic settings for precursor and product ion tolerance, a minimum of 1 peptide matches per protein, with a minimum of 5 consecutive fragment ions per peptide, and a minimum number of product ion matches per protein, 7.

### *Phaedon* cDNA library generation and sequencing

As the genome of *Phaedon* is not yet characterized, a dual approach was taken towards sequencing in order to balance costs and the ability to *de novo* assemble the sequence data, combining both Sanger and 454 sequencing of normalized cDNA libraries and RNASeq of selected samples. *Phaedon cochleariae* total RNA was isolated from all developmental stages and a wide range of tissues and treatments, including larvae and adult beetles that had been exposed to phytochemicals, different host plants, starvation, and immune induction experiments using a combination of bacteria and fungi.

In order to avoid over-representation of the most commonly transcribed genes, full-length enriched, normalized cDNA libraries were generated using a combination of the Mint-Universal cDNA synthesis kit (Evrogen, Moscow, Russia) and the Trimmer Direct cDNA normalization kit (Evrogen). The procedure generally followed the manufacturer’s protocol but included several important modifications,as described [57]. Optimization of the complete cDNA normalization procedure was performed as described in [58]. The resulting normalized cDNA library was used for 454 pyrosequencing using the Roche 454 FLX machine and Sanger sequencing on an ABI 3730 xl automatic DNA sequencer (PE Applied Biosystems). The 454 sequence reads were assembled using the CLC Genomics Workbench (CLC bio version 5.0.1; http://www.clcbio.com). Adaptors were removed, and sequences were trimmed for length and quality with standard settings. Assembly was performed using the standard CLC parameters for long reads. Contigs shorter than 250bp were removed from the final analysis. A fraction of the ds-cDNAs was cloned in the pDNR-Lib vector (Clontech). Bacterial transformation, plasmid minipreparation, single-pass sequencing of cDNA library clones and sequence assembly were performed as described in [59].

### RNASeq data generation, assembly and annotation

RNASeq was performed with dissected larvae and adult beetles, resulting in four sample pools: adult guts, adult rest body, larval guts and larval rest body. Three days before RNA extraction, insects from all developmental stages were placed on Chinese and white cabbage plants. Larvae of all three instars as well as adults of both sexes were dissected. Guts and the rest of the bodies were separately stabilized in RNA*later* solution (Qiagen) and stored at −20°C. Total RNA isolations were performed using the RNeasy Micro Kit (Qiagen) following the manufacturer′s guidelines. Integrity and quality of the RNA samples were determined using the RNA 6000 Nano LabChip kit (Agilent Technologies) on an Agilent 2100 Bioanalyzer (Agilent Technologies) according to the manufacturer’s instructions.

RNAseq was outsourced to Fasteris ( http://www.fasteris.com), using 5 μg total RNA isolated from the four samples described above. RNASeq was performed using the HiSeq^TM^ 2000 Sequencing System from Illumina ( http://www.illumina.com/), utilizing the single read 100 bp technology. CLC Genomics Workbench was used for sequence assembly of the resulting 75 Mio sequence reads. First, sequences were trimmed for length and quality with standard settings and subsequently assembled using the following CLC parameters: nucleotide mismatch cost = 2; insertion=deletion costs = 2; length fraction = 0.3; similarity = 0.9. Any conflicts among the individual bases were resolved by voting for the base with highest frequency. Contigs shorter than 250bp were removed from the final analysis. The Sanger, 454 and Illumina assemblies were subsequently reassembled using the SeqMan assembly tool (98% sequence identity cutoff, 45bp overlap) implemented in the Lasergene software package (DNAStar Inc.), resulting in a final *de novo* reference assembly (backbone) of 63,115 contigs and singletons. The digital gene expression analysis was performed with QSeq Software (DNAStar Inc.), utilizing the respective mapping tools by mapping each Illumina sequence to the obtained reference backbone sequences, which was then used to estimate expression levels. The correction for biases in the sequence datasets and different transcript sizes were addressed using the RPKM (reads per kilobase of transcript per million of reads sequenced) algorithm to obtain correct estimates of relative expression levels. Homology searches (BLASTx and BLASTn) of unique sequences and functional annotation by gene ontology terms (GO; http://www.geneontology.org), InterPro terms (InterProScan, EBI), enzyme classification codes (EC), and metabolic pathways (KEGG, Kyoto Encyclopedia of Genes and Genomes) were determined using the BLAST2GO software suite v2.4.1 ( http://www.blast2go.de).

### Manual curation of cDNAs and full length sequencing

Contigs corresponding to sequences of interest were retrieved from the transcriptome database. Sequences of cDNAs encoding full-length transcripts were confirmed by designing specific primers used to re-amplify the complete open reading frame (ORF). cDNA sequences encoding only a partial ORF were used to design specific primer pairs to perform 5’- and 3’-Rapid Amplification of cDNA ends (RACE) PCRs. For these we used the SMARTer RACE cDNA Amplification Kit (BD Clontech) according to the manufacturer’s instructions. PCR products were cloned into TOPO TA 2.1 vector (Invitrogen) for sequencing. All cDNA sequences encoding ORFs were annotated and submitted to EMBL under accession numbers HE962191 to HE962210.

### Analysis of PCWDE expression

For qPCR, early third-instar larvae were reared on Chinese cabbage plants for 24 h prior to dissection. Guts and the rest of the bodies were dissected and 500 ng of total RNA pooled from the tissues of 10 larvae was reverse-transcribed with a 3:1 mix of random and oligo-dT20 primers. Real-time qPCR was performed in optical 96-well plates on a Stratagene MX 3000P system. All steps were performed with the Verso SYBR Green 2-Step QRT-PCR Kit Plus ROX Vial (Thermo Scientific) following the manufacturer′s instructions. The specific amplification of transcripts was verified by dissociation curve analysis. All primers were designed using Primer3 (version 0.4.0). *Eukaryotic initiation factor* 4A (HE962192) and *elongation factor* 1α (HE962191) were used as reference genes to normalize quantities of the genes of interest. Raw data were analyzed with qBase, using the fold change of relative expression levels in the guts compared to the rest of the bodies on the y-axis of the graphs, with lower transcript abundance (in the rest of the bodies) set to 1.

## Competing interests

The authors declare that they have no competing interests.

## Authors' contributions

RK, DGH and YP designed the research. RK and YP performed the proteomics experiments. NW and AS performed the mass spectrometry analyses and the database searches. RK and HV generated the transcriptome data. RK performed the quantitative PCR experiments. HV performed the RNA-SEQ analysis. RK, HV and YP analyzed the data. YP and DGH wrote the manuscript. All authors read and approved the final manuscript.

## Supplementary Material

Additional file 1 **Figure S1.** Chromatogram of the anion exchange chromatography of *P. cochleariae* gut content proteins; Figure S2, CMC and pectin zymograms; Figure S3 , GH11s amino acid alignment; Figure S4, GH28s amino acid alignment; Figure S5, GH45s amino acid alignment; Table S1, possible N-glycosylation sites found for each putative *P. cochleariae* PCWDE. Click here for file

Additional file 2 **Details of the protein identifications obtained from the proteomics approach after mass spectrometry analyses.** For each analysis performed (LC-MS/MS or LC-MS^E^, searching either against a viridiplantae of the *P. cochleariae* database), data are presented in a separated spreadsheet. Click here for file
