# Supplementary material for: Combining proteomics and transcriptome sequencing to identify active plant-cell-wall-degrading enzymes in a leaf beetle
Source: BMC Genomics. 2012 Nov 1;13:587. doi: 10.1186/1471-2164-13-587 (PMC3505185; doi:10.1186/1471-2164-13-587)
Supplement: Additional file 1 — Figure S1. Chromatogram of the anion exchange chromatography of P. cochleariae gut content proteins; Figure S2, CMC and pectin zymograms; Figure S3 , GH11s amino acid alignment; Figure S4, GH28s amino acid alignment; Figure S5, GH45s amino acid alignment; Table S1, possible N-glycosylation sites found for each putative P. cochleariae PCWDE. [file 1471-2164-13-587-S1.doc]

**Figure S1.** Resulting chromatogram of *P. cochleariae* gut content proteins separated by anion exchange chromatography. The UV trace, reflecting the presence of proteins, is shown in blue. The fraction numbers recovered are indicated in dark green. The light green line represents the 0 to 1 M linear NaCl gradient.

**Figure S2.**  *Phaedon cochleariae* gut contents exhibit activity towards cellulose and pectic acid by zymogram assays. Five microliters of each fraction from the anion exchange chromatography were resolved on a 12.5 % SDS-PAGE gel containing either 0.1 % carboxymethylcellulose (A) or 0.1 % pectic acid from citrus peels (B). Enzymatic activity is represented by pale bands on a dark background. No activity was detected using xylan and galactomannan as substrates (not shown).


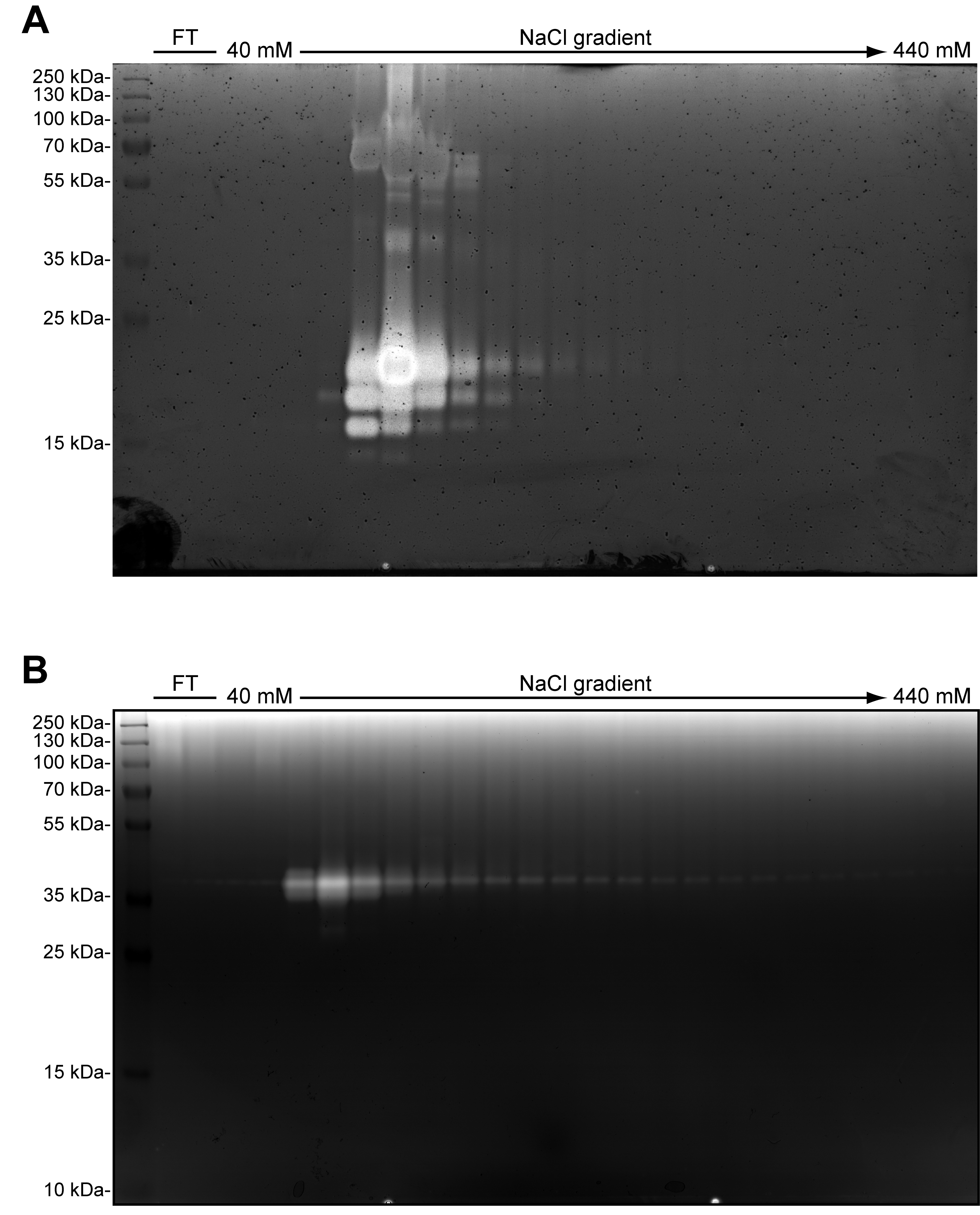


**Figure S3.** Amino acid alignment of the two putative GH11 xylanases found in the *P. cochleariae* transcriptome. Amino acid alignment was performed using the MUSCLE program. Putative signal peptides (first 17 amino acids) are indicated by an arrow. The putative catalytic residues are indicated by arrowheads. Peptides from the proteomic analysis matching GH11-1 and GH11-2 are indicated in red and in green respectively.


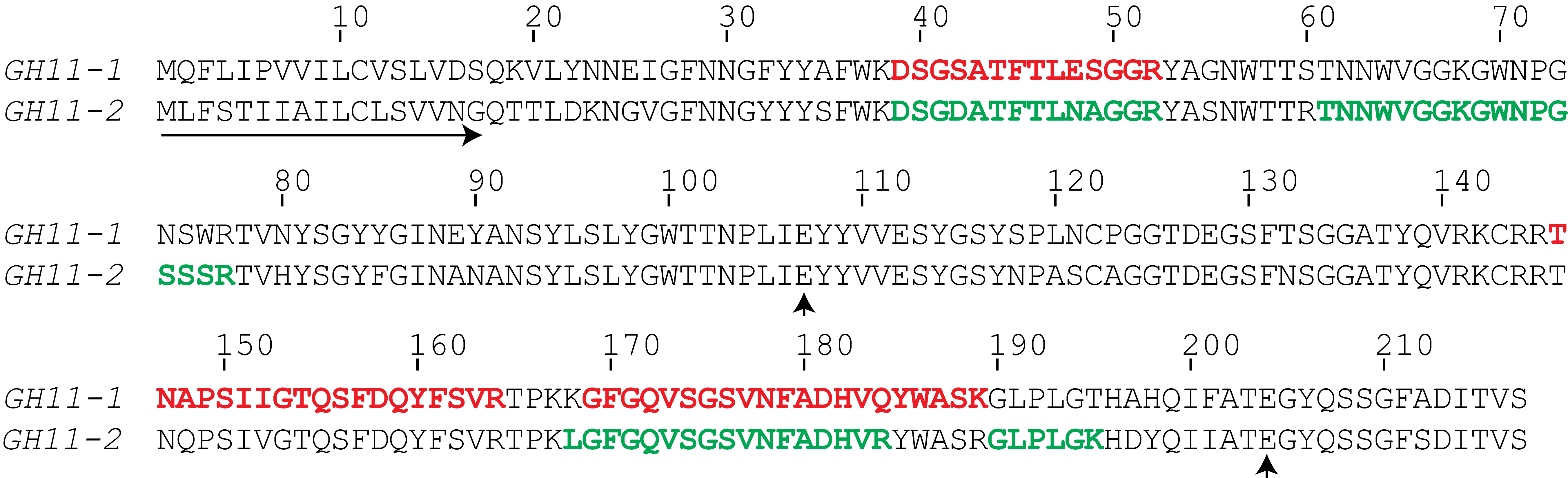


**Figure S4.** Amino acid alignment of the putative nine GH28 polygalacturonases found in the *P. cochleariae* transcriptome. Putative signal peptides were removed from the sequences prior to alignment using the MUSCLE program (A). A Neighbor-Joining relationship of the corresponding sequences is also presented (B). Branch support was tested by bootstrap analysis using 1,000 replicates. Values above 40 are indicated next to the branches. The putative catalytic residues are indicated by an arrow. Peptides from the proteomic analysis matching GH28-1, GH28-3, GH28-6, GH28-7 and GH28-9 are indicated in red.


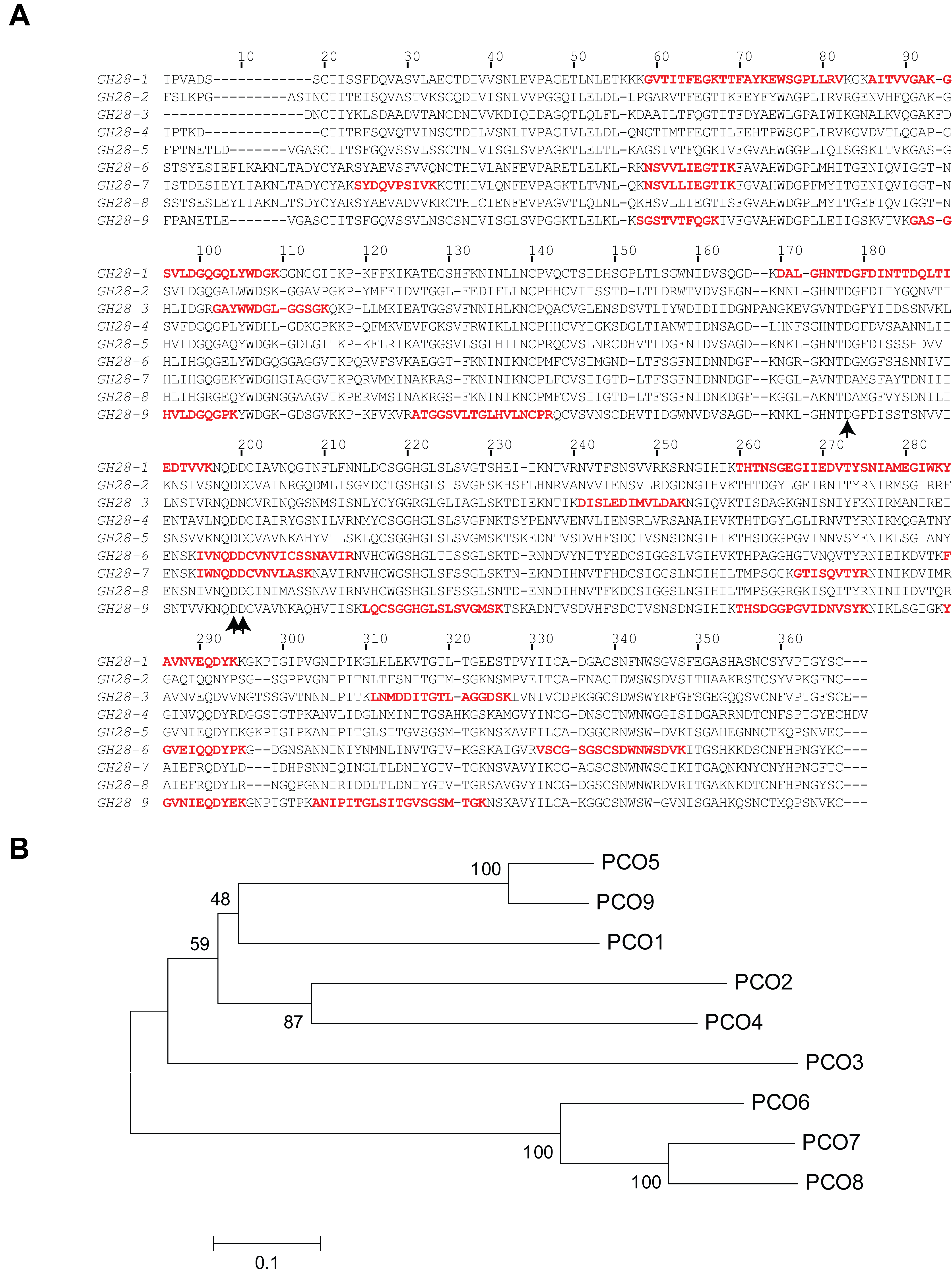


**Figure S5.** Amino acid alignment of the seven putative GH45 cellulases found in the *P. cochleariae* transcriptome. Putative signal peptides were removed from the sequences prior to alignment using the MUSCLE program (A). The degree of conservation of each residue is represented by shading going from dark (conserved residue) to white (non-conserved residue). A Neighbor-Joining relationship of the corresponding sequences is also presented (B). Branch support was tested by bootstrap analysis using 1,000 replicates. Values above 50 are indicated next to the branches. The putative catalytic residues are indicated by red boxes.


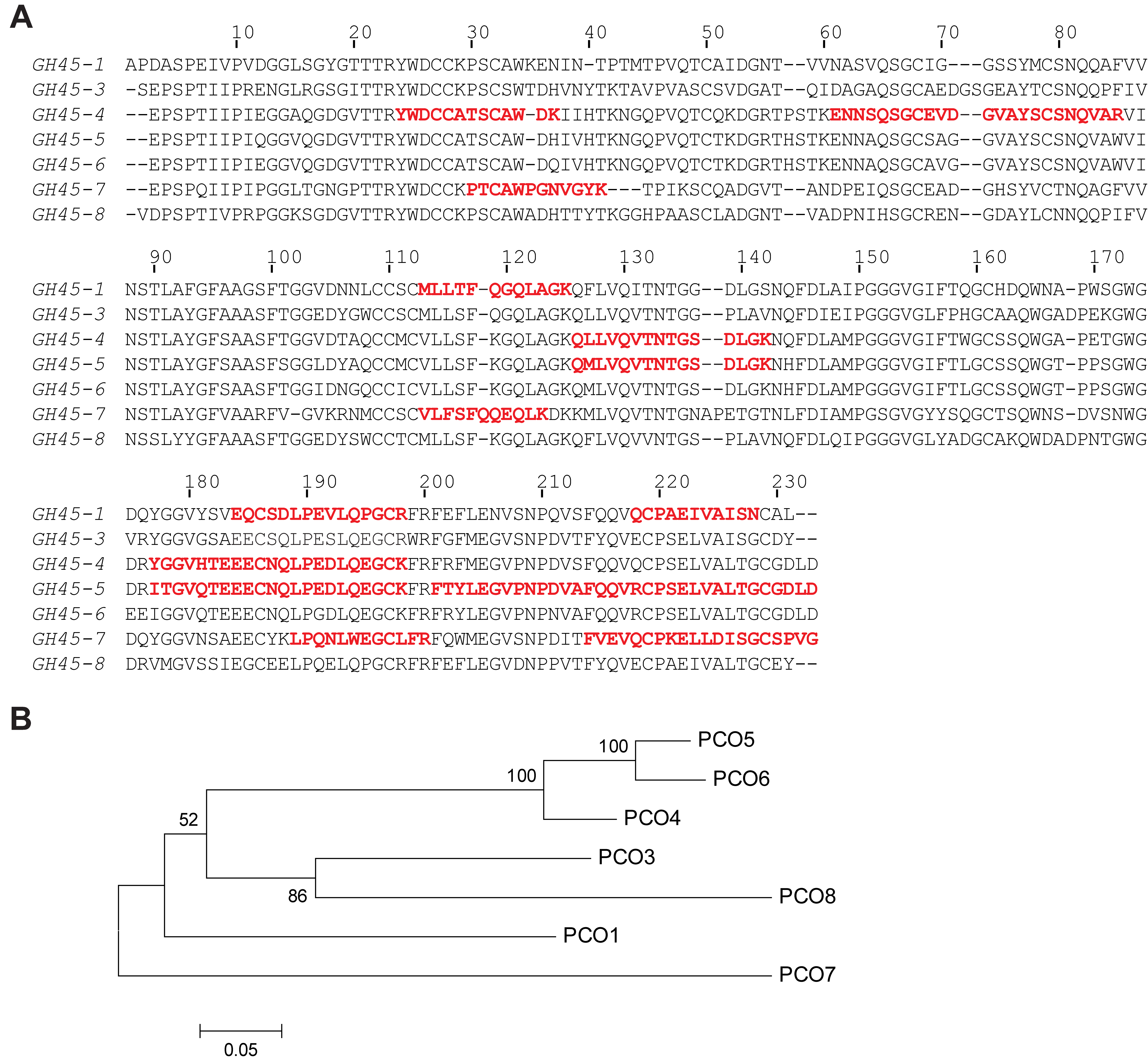


**Table S1.** Predicted number of N-glycosylation sites for every *P. cochleariae* putative PCWDE.

|  | GH11 | | GH28 | | | | | | | | | GH45 | | | | | | |
| --- | --- | --- | --- | --- | --- | --- | --- | --- | --- | --- | --- | --- | --- | --- | --- | --- | --- | --- |
| ID | 1 | 2 | 1 | 2 | 3 | 4 | 5 | 6 | 7 | 8 | 9 | 1 | 3 | 4 | 5 | 6 | 7 | 8 |
| N-Glyc sites | 2 | 1 | 3 | 4 | 5 | 7 | 3 | 4 | 3 | 2 | 4 | 3 | 2 | 1 | 1 | 1 | 1 | 1 |
